# Information Seeking Practices and Availability of Smart Devices Among Healthcare Professionals in a Lower-Middle-Income Country: An Analysis From Pakistan

**DOI:** 10.7759/cureus.30771

**Published:** 2022-10-27

**Authors:** Sufyan Razak, Shilpa Bai, Faiza Zakaria, Mustafa Ali, FNU Rashmi, Reem Sulaiman, Ayesha Altaf Jangda, Ashish Tyagi, FNU Nima, Syed Asad Hasan Rizvi

**Affiliations:** 1 Internal Medicine, Dow University of Health Sciences, Karachi, PAK; 2 Oncology, Johns Hopkins University School of Medicine, Baltimore, USA; 3 Pathology and Laboratory Medicine, Jinnah Sindh Medical University, Karachi, PAK; 4 Oncology, Dow University of Health Sciences, Karachi, PAK; 5 Internal medicine, Jinnah Sindh Medical University, Karachi, PAK; 6 Internal Medicine, Sharif Medical and Dental College, Lahore, PAK; 7 Internal Medicine, Ziauddin University, Karachi, PAK; 8 Pathology and Laboratory Medicine, Johns Hopkins University School of Medicine, Baltimore, USA; 9 Internal Medicine, Jinnah Sindh Medical University, Karachi, PAK

**Keywords:** computer technology, pubmed database, smart devices, iphone, android, mobile phones, lower middle income country, internet, evidence based medicine, medical education

## Abstract

Introduction

Evidence-based medicine (EBM) is a principle that integrates clinical experience with relevant information available to provide adequate healthcare. It requires access to current medical literature. This paper analyzes the information requirements of a lower-middle-income country (LMIC) and the resources available and preferred by medical professionals.

Methods

A survey-based cross-sectional study was carried out among 160 participants, ranging in expertise from students to attending physicians in Karachi, Pakistan. The survey comprised questions to assess the clinical background, technology access, need for health-related information, and the preference for resources to obtain that information in different scenarios. They were also asked if they use PubMed and their recommended methods to improve information access. Statistical Package for the Social Sciences (SPSS; IBM, NY, USA) software was used for all analyses.

Results

A basic mobile phone (with limited internet connectivity) was the most common device used at home (n=159; 99.4%) and work (n=141; 88.1%). No smart devices were available to 28 (17.5%) participants at work. Internet connectivity was available for 155 (96.9%) participants at home but only for 118 (73.7%) participants at work. About one-third (n=49; 30.6%) experienced questions arising in practice two to four times a day, and half of the participants (n=80; 50%) were very likely to look up a reference. The most common resource for the majority of given clinical scenarios was a senior colleague. At the same time, medical websites (Medscape, Up-to-Date, WebMD) were the first choice for a non-specific general medical query. About 68.75% (n=110) of participants claimed to use PubMed in daily practice. The most common reason for not using PubMed was the ease of using other search engines (like Google).

Conclusions

Improved access to the internet and well-reputed journals can enhance the practice of EBM in Pakistan. Limitations of technological access must be considered while designing information resources in lower-middle-income countries.

## Introduction

Evidence-based medicine (EBM) integrates crucial components that are fundamental in providing quality healthcare: the physician’s expertise, the patient’s inclination, and most importantly, scientific research [1]. With constant evolution in the world of medicine, timely access to appropriate advancements in the field is essential in shaping a clinician’s response to patient management queries. The internet and technological developments have assisted physicians colossally in achieving the goal. Access to healthcare information is not limited to medical personnel and has extended significantly to patients as well, assisting them in better understanding their ailments [2].

In the developing world, access to online medical information is far more limited compared to the developed world. Statistics show a penetration rate of 63.8% in Asia, compared to a rate of 93.9% in North America [3]. Significant data have been published regarding information-seeking behavior in developed countries [4-7]; however, this data is scarce for developing countries [8], with very few focusing solely on South Asia [9]. Studies from high-income countries show the utilization of electronic sources, medical libraries, treatment guidelines, and scientific journals to solve medical queries, and discussions with colleagues [10-14]. Owing to a dearth of resources and lack of accessibility, this practice is bound to differ among students and clinicians of low-income countries, amounting to significant differences in information-seeking strategies. Textbooks have been identified as a primary source of information, in addition to consultation with colleagues [8]. However, the latter strategy is found to overlap in Western Countries as well, owing to poor search strategies, geographical and time limitations, and inadequate access to electronic devices [15]. In contrast, this behavior in developing countries could be owed to a lack of technologies to access information [16]. A study from the Philippines reported that about 82% of physicians did not have a basic mobile phone at work. Laptops/computers were available only to 73% of participants, and internet connectivity was accessible only to 46% of participants at work, making information less accessible [16].

In this study, we sought to fill a pertinent gap by assessing the clinical background, technology access, need for health-related information, and preference of resources to obtain medical information among the medical workforce, alongside students of Karachi, Pakistan. Additionally, we inquired about PubMed use and their recommendations to improve this discrepancy. This assessment could prove to be a stepping stone in improving access to medical information and improving EBM practice and patient outcomes, which may apply to low-income countries.

## Materials and methods

Study design, duration, and population

A descriptive cross-sectional study was conducted in February 2019 for one month. A non-probability self-selection sampling method was adopted for the study. The study population consisted of individuals from Karachi who were associated with the medical profession and accepted to participate in our survey. This included medical students, house officers, resident physician trainees, general or community health practitioners, consultants, and specialists. The participation was completely voluntary, and formal consent was obtained prior to the study.

The survey did not address patients or animals and no clinical experiments or records were included in this study. The authors declare that the work described has been carried out in accordance with the Declaration of Helsinki of the World Medical Association revised in 2013 for experiments involving humans. No identifiable information was obtained, and all data were non-identifiable. Ethical approval was not required for this survey of health professionals as it was assessed to have no physical and psychological harm and ethical risk according to current recommendations [16,17]. All participants were independent competent adults that voluntarily filled out the survey and had complete authority to withdraw from the study at any time.

Details of questionnaire

With permission (from Dr. Paul Fontelo), we modified a questionnaire used in a study conducted in the Philippines by Gavino et al. [16]. The initial questionnaire was modified to match our study's target population and objectives. The questionnaire consisted of 17 questions to assess the clinical background, technology access, need for health-related information, and the preference for resources to obtain that information.

To evaluate their clinical background, we asked the participants about their clinical practice and specialty description. To assess the technology access, we asked the participants if they availed of the following facilities at home and work: (1) Basic mobile phone (which has basic features of SMS and MMS, Voice call with no or limited Internet access); (2) Smart devices (Android phone, iPhone, Blackberry, Windows phone, or tablet device); (3) Computer (laptop or desktop); (4) Internet connectivity; and (5) Messaging service and modality (SMS, e-mail, MMS, Instant messaging with WhatsApp, Viber, Facebook Messenger, and video calling with WhatsApp, Skype, etc.).

To assess the need for health-related information in their practice, we asked the participants about the frequency of health-related questions in their practice, how likely they are to search for their answers, and where they would search for them. We presented six clinical scenarios to the participants to assess their preferred resources to obtain health-related information. Each raised a question regarding diagnosis, management, drug information, prognosis, information resource recommendation to the patient, and general medical inquiry. The participants were asked to choose their top three preferred information resources from 14 different choices in each scenario. We then asked the participants to mention three medical journals that they found the most useful in their clinical practice. We also asked the participants if they used PubMed or MEDLINE in their practice and why they did not. Finally, we asked the participants for recommendations to improve healthcare information access to medical professionals.

Data collection

The questionnaire was distributed to the study population in two forms, an online version, and a printed paper version. The online version was distributed through E-mails, professional networks, and social media. The printed copy of the questionnaire was distributed in person in grand rounds, ward morning meetings, medical school classes, outpatient departments, and seminars. A total of 160 individuals participated in the study for one month.

Statistical analysis

All the data were entered into a preformed data sheet based on the questionnaire variables. The variables were divided into categorical and numerical variables. All the data was then analyzed using Statistical Package for the Social Sciences (SPSS) version 17.0 (IBM, NY, USA). Frequencies were calculated using descriptive statistics. Tables and graphs were generated using Microsoft Excel. Percentages and their corresponding numbers were reported in tables.

## Results

A total of 160 individuals participated in the study. Among these, 98 (61.3%) filled out the printed paper version of the questionnaire, while 62 (38.8%) submitted their responses online.

Clinical background

Table 1 describes the clinical background of the participants. Most participants were medical students, followed by general health practitioners working in government hospitals and house officers.

**Table 1:** Clinical background of participants. The table shows the number and percentages of the different clinical backgrounds of participants.

Clinical background	Frequency (n)	Percentage (%)
Medical student	82	51.3
Primary care/ general practice in a government hospital	20	12.5
House officer	16	10.0
Resident physician training in a government hospital	13	8.1
Primary care/ general practice in a private hospital / clinic	12	7.5
Specialist/ consultant/ attending in a government or private hospital / clinic	10	6.3
Resident physician training in a private hospital	5	3.1
Community/ public health practitioner	2	1.3

**Figure 1:**
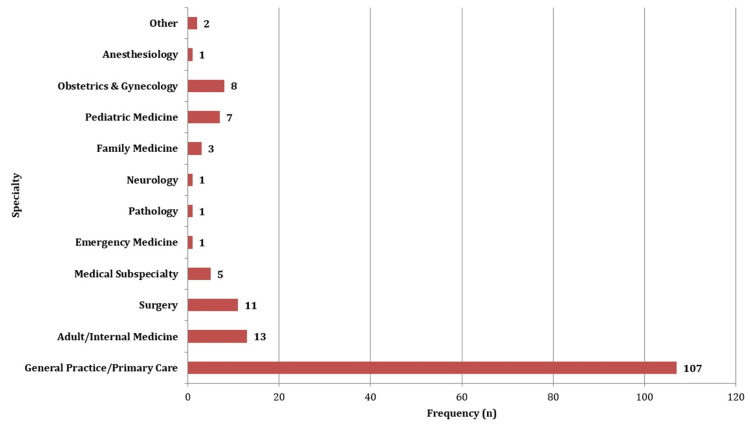
Specialty profile of participants.

Figure 1 displays the specialty profile of the participants. Most of the participants were associated with general practice or primary care (66.9%), followed by internal medicine (8.1%) and surgery (6.9%).

Technology access

The majority of the participants had a basic mobile phone at home as well as at work. In both settings (at home and work), most participants used Android phones, followed by iPhones, and only one participant reported having a Blackberry phone. No smart devices were available to 28 (17.5%) participants at work. Most of the participants (n=147; 91.9%) reported having all the facilities, including SMS, e-mail, MMS, instant messaging (WhatsApp, Viber, Facebook messenger, etc.), and video calling (WhatsApp, Skype, etc.) at home.

At work, almost half (n=73; 45.6%) of participants reported having no computer available, and an internet connection was not available to 42 (26.3%) participants. The details of technology access to participants are given in Table 2.

**Table 2:** Details of technology access to participants SMS, short message service; MMS, multimedia messaging service

Technology/ devices	Availability	
	At home, n (%)	At work, n (%)
Mobile		
Basic mobile phone	159 (99.4)	141 (88.1)
Android phone	100 (62.5)	89 (55.6)
iPhone	58 (36.2)	39 (24.4)
Windows phone	01 (0.6)	1 (0.6)
Blackberry	0 (0.0)	1 (0.6)
Tablet device	0 (0.0)	2 (1.3)
Communication services		
SMS	147 (91.9)	109 (68.1)
MMS	147 (91.9)	98 (61.3)
E-mail	151 (94.4)	104 (65.0)
Instant messaging (WhatsApp, Viber, Facebook messenger, etc.)	152 (95.0)	99 (61.9)
Video calling (WhatsApp, Skype, etc.)	147 (91.9)	99 (61.9)
Other facilities		
Computer (laptop or desktop)	150 (93.8)	87 (54.4)
Internet connectivity	155 (96.9)	118 (73.8)

Clinical information needs

We asked the participants about how often they encountered questions in their practice that compelled them to look up a reference source for answers. Most participants (n=49) reported facing such questions 2-4 times a day (30.6%). Slightly less (n=42) encountered them once daily (26.3%), 36 (22.5%) rarely, 23 (14.4%) more than five times daily, while seven said that they usually did not face any questions (4.4%). Three participants (1.9%) did not answer our question.

Facing these clinical questions, 80 participants (50%) said they were very likely to look up a reference source, 29 said that they were somewhat unlikely to do it (18.1%), 26 (16.3%) neither likely nor unlikely, while 20 said they always looked up a reference source for answers to these questions (12.5%). The rest of the five participants said they would never look up a reference source when such questions arose (3.1%).

**Figure 2:**
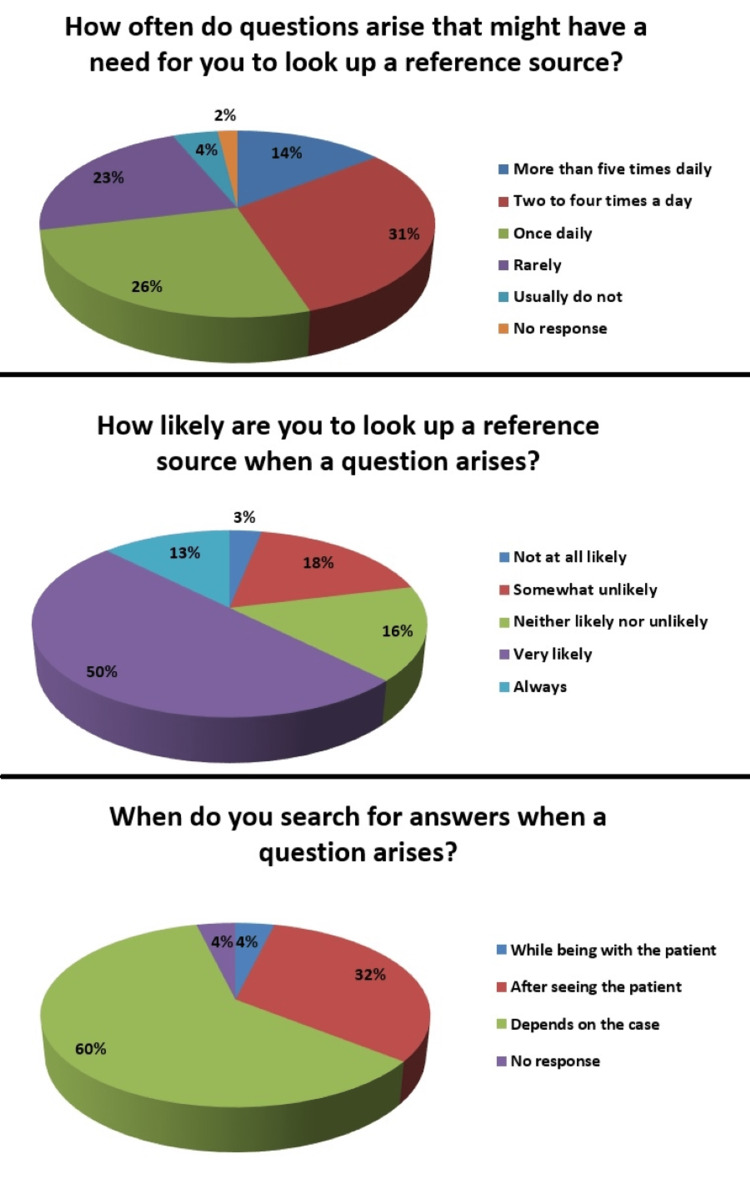
Responses of participants about clinical information needs scenarios The questions along with their responses and relevant percentages are reported in the figure.

We then asked the participants about when they would search for the answers to these questions. For most of the participants (n=97, 60.6%), it depended upon the individual case. Fifty-one said they would look for answers after seeing the patient (31.9%), while six said they would do so while with the patient (3.8%). Six participants did not answer our question (3.8%). The responses are summarized in Figure 2.

Information preference

We then gave six clinical scenarios to the participants and asked about their preferred source of information. Table 3 describes the most frequently opted sources of information for each clinical scenario.

**Table 3:** Frequently opted sources of information for each clinical scenario The table shows the source of information health care professionals preferred in different scenarios. First, second, and third indicate their preference in ascending order.

Scenario about:	The preferred source of information from healthcare professionals		
	First	Second	Third
Drug indications and dosage	Senior colleague/attending/consultant	Hard-copy of book, textbook, or journal	Medical websites (Medscape, Up-to-Date, WebMD, etc.)
Diagnostic tests and workup	Senior colleague/attending/consultant	Medical websites (Medscape, Up-to-Date, WebMD, etc.)	Search engine (Google, Yahoo, Wikipedia, etc.) Medical Smartphone applications (Medscape, PubMed Mobile, etc.)
Therapeutic management	Senior colleague/attending/consultant	Hard-copy of book, textbook, or journal	Medical websites (Medscape, Up-to-Date, WebMD, etc.)
Prognosis	Senior colleague/attending/consultant	Medical websites (Medscape, Up-to-Date, WebMD, etc.)	Search Engine (Google, Yahoo, Wikipedia, etc.)
General medical question	Medical websites (medscape, up-to-date, webmd, etc.)	PubMed / Medline	Search Engine (Google, Yahoo, Wikipedia, etc.)
Recommendation to patients	Senior colleague/attending/consultant	Medical websites (Medscape, Up-to-Date, WebMD, etc.)	Search Engine (Google, Yahoo, Wikipedia, etc.)

Most of the participants were unaware of any journal names that were relevant to their practice. Among the journals that the participants mentioned, the most popular choice was the Journal of Pakistan Medical Association (JPMA), mentioned by 19 participants (11.87%).

PubMed use

When asked if they used PubMed or MEDLINE in their practice, most of the participants (n=110; 68.75%) admitted using it, while 50 (31.25%) participants did not. We asked these 50 participants the reason for not using PubMed or MEDLINE, the most popular reason stated by the participants was the ease of using internet search engines such as Google (50%), followed by the lack of awareness of these tools (26%). Most participants (n=125; 78.1%) reported free or open Internet access was necessary to improve healthcare information access for medical professionals.

## Discussion

Evidence-based medicine

EBM has become an indispensable tool in the clinical decision-making process. Over the past couple of decades, multiple studies have demonstrated the landmark differences in patient care by incorporating appropriate technologies, which allow easy accessibility to medical knowledge and resources in the clinical setting [8,18,19]. In this study, we assessed the information requirements of a Lower-Middle Income Country (LMIC), the resources available, and those preferred by medical professionals.

Technology access

Our results showed that basic mobile phones (with limited internet connectivity) are the most common device used at work (88.1%), with no smart devices being available to 17.5% of participants. Moreover, 26.3% of participants did not have Internet connectivity available at work. About 71.3% of the participants encountered clinical questions at least once daily, of which 62.5% were likely to look up a reference. An analysis of available literature revealed similar findings in another LMIC [16]. A study from the Philippines reported that 82.19% of participants had a basic mobile phone, 13.01% had iPhones, 7.53% had Blackberry phones, 6.16% had tablet devices, and 4.79% had Android phones. Laptops were available to 92.47% of participants at home, and 72.60% had them at the workplace. Only 45.89% of the participants reported having internet access while at work [16].

Our findings denote that only 50% of Pakistan's doctors are likely to investigate a clinical query by looking up references. Similarly, a study conducted in the Philippines demonstrates that 58% of the participants were likely to utilize technology to answer their clinical questions [16]. For information regarding drug indications and dosages, diagnostic workup, therapeutic management, prognosis, and recommendations to the patient, in our study, doctors were most likely to rely on their senior colleagues, attending, or consultants. However, in the survey conducted by Gavin et al., for the concerns mentioned above, participants were most likely to rely on printed copies of formularies (for drugs and dosages), colleague or librarian (for diagnostic workups), medical websites (for therapeutic management), PubMed (for prognosis) and disease-specific organization website (for patient recommendations), respectively [16]. Additionally, the preferred source of general medical information for doctors in the Philippines remained PubMed [16]. In our study, participants were more likely to rely on Medscape, Up-to-date, and WebMD websites. Similar to our research, search engines such as Google ranked third for general medical questions. Our findings indicate that the most likely reason (50%) for not utilizing the PubMed database remains the feasibility of search engines such as Google, followed by a lack of awareness of these tools (26%), whereas, in the Philippines, the number one cause remains lack of awareness [16].

Shifting trends

Over the past years, the trend has markedly shifted from using books to accessing medical information online resources [20]. This is especially true for developed countries where access to the Internet and smart devices distinguish them from LMICs, which have been lagging [21].

Research has shown that quick access to medical information over the Internet can significantly improve patient care [22]. This study demonstrated that 26.3% of professionals did not have access to the internet at their workplace, which prevented them from being able to look up pertinent medical information. Moreover, recent statistics have shown Internet penetration to be substantially lower in LMICs such as Pakistan when compared to the developed world [3,23].

The smartphone is an exceptionally important piece of this puzzle. Recently, widespread access to smart devices has made it effortless to look up pertinent questions that healthcare professionals might have [24]. Not only does this reduce errors in patient care resulting from prescribing medication, but it also improves compliance with international practice guidelines [22]. Nowhere was this more prominent than during the ongoing COVID 19 when access to frequently changing guidelines became vital to effective patient management [25].

Information preferences

According to our results, the single most preferred source of information was a senior/colleague, with online sources such as PubMed being second. This might be due to the significantly greater amount of time that it takes to access electronic information, as identified by a previously conducted study. Additionally, the lack of the ability to formulate a good question and interpret the results may be a significant contributory factor [7].

When asked if they used PubMed in their practice, most of the participants (n=110; 68.8%) admitted to using it; this finding was comparable with a previously conducted study in the Philippines [16]. This is in contrast with the fact that most participants (n=88; 13%) were unfamiliar with even one journal relevant to their fields. This may be attributable to the lack of focus on research practices in LMICs medical schools [26].

Recommendations and limitations 

Recent studies have shown that using supplementary smart devices, including laptops and tablets, can significantly impact medical education [27,28]. Therefore, technological resources, such as a 3D atlas and online video lectures, are recommended to be built into the medical school curriculum. Students are encouraged to review international guidelines from an earlier point in their careers. Moreover, physicians should have Internet-connected devices during patient follow-up, allowing them to quickly check any relevant medical questions during or even between patient visits, ensuring patients can receive up-to-date and evidence-based treatments. Furthermore, it is vital that physicians can access recent medical literature, therefore in LMIC where such journals are often unaffordable, free open access should be provided for physicians. EBM should be promoted for a uniform standard of care and patient safety. Some mobile applications allow offline reading, and users can benefit from them even without internet access.

Using technology to acquire knowledge of evidence-based treatments and workups will be paramount in diagnosing and treating medical diseases. A few reasons can be speculated as the cause of the underutilization of technology in LMIC. First, the lack of access to such resources due to financial restraints remains a limitation. Secondly, very often, LMIC physicians who belong to an older generation may resort to orthodox practices of referring to textbooks or a colleague instead of turning to the internet. This may also be compounded by the senior physicians' difficulty operating technological devices, which is a significant hindrance. A separate study is needed to implore the possible causes of the underutilization of technology in LMIC.

It is also important to identify some limitations in our study. The major one is that it was conducted at a single tertiary care teaching hospital. Multi-center studies need to be conducted on a larger scale in both public and private setups and other parts of the country to give a more holistic perspective of the current situation in LMICs. Moreover, data were collected from professionals across multiple specialties. Further stratification may reveal trends specific to certain subspecialties.

## Conclusions

To promote the practice of evidence-based medical care in LMICs such as Pakistan, access to the internet is of the utmost importance. Additionally, access to medical literature on the web at a subsidized amount or free of cost could serve to advance the culture of EBM in Pakistan. However, technological limitations must be considered during this process, with propositions to modify these hindrances. The ease of availability of computers and the internet can enhance the exposure of physicians to recent medical developments. Journal reading and use of EBM should be promoted at all medical education levels.
